# Vom Bauchgefühl zum viszeralen Schmerz: Effekte negativer Erwartungen im Kontext der Darm-Gehirn-Achse

**DOI:** 10.1007/s00482-021-00614-w

**Published:** 2021-12-23

**Authors:** Jana Aulenkamp, Kathrin Steinmüller, Adriane Icenhour, Sigrid Elsenbruch

**Affiliations:** 1 Klinik für Neurologie, Universitätsklinikum Essen, Hufelandstraße 55, 45147 Essen, Deutschland; 2 Klinik für Anästhesiologie und Intensivmedizin, Universitätsklinikum Essen, Hufelandstraße 55, 45147 Essen, Deutschland; 3 Institut für Medizinische Psychologie und Medizinische Soziologie, Ruhr Universität Bochum, Universitätsstraße 150, 44801 Bochum, Deutschland

**Keywords:** Noceboeffekt, Chronischer Schmerz, Hypervigilanz, Schmerzbezogene Furcht, Psychologische Faktoren, Nocebo effect, Chronic pain, Hypervigilance, Fear, pain-related, Psychological factors

## Abstract

Störungen der Darm-Gehirn-Achse sind durch komplexe Dysfunktionen auf peripherer und zentralnervöser Ebene gekennzeichnet, die zu viszeraler Hypervigilanz und Hyperalgesie beitragen können und viszeralen Schmerz prägen. An der viszeralen Schmerzmodulation sind zahlreiche kognitive, emotionale und psychoneurobiologische Faktoren beteiligt, die im psychosozialen Therapiekontext das viszerale Schmerzerleben sowohl positiv als auch negativ beeinflussen können. Durch negative Erwartungen vermittelte Noceboeffekte sind bei akuten, aber insbesondere auch bei chronischen viszeralen Schmerzen von hoher klinischer Relevanz; die ihnen zugrunde liegenden Mechanismen sind jedoch bislang unzureichend verstanden. Zur Entstehung und Aufrechterhaltung negativer Erwartungseffekte tragen insbesondere verbale Instruktionen, Vorerfahrungen und Lernprozesse sowie emotionale Faktoren wie Angst und Stress bei. Gezielte Kommunikationsstrategien, ein sensibler Umgang in der Aufklärung und positive Umgebungsbedingungen können in der klinischen Praxis dazu beitragen, ein adäquates Erwartungsmanagement zu etablieren und negative Erwartungseffekte zu minimieren. Zugleich sind translationale Forschungsansätze erforderlich, um tiefere Erkenntnisse bezüglich der Mediatoren und Moderatoren negativer Erwartungseffekte zu erlangen und diese in die Klinik zu übertragen. So kann die Versorgung von Patienten mit Störungen der Darm-Gehirn-Kommunikation verbessert werden.

## Abstract (en)

Disturbances of the gut-brain axis are characterized by complex dysfunctions on peripheral and central nervous system levels, which can contribute to visceral hypervigilance and hyperalgesia and imprint visceral pain. Numerous cognitive, emotional and psychoneurobiological factors are involved in visceral pain modulation, which in the psychosocial treatment concept can have a positive as well as a negative impact on the experience of visceral pain. Nocebo effects induced by negative expectations are of high clinical relevance in acute and especially in chronic visceral pain but the underlying mechanisms remain insufficiently understood. Verbal instructions, previous experiences and learning processes as well as emotional factors, such as fear and stress contribute to the development and maintenance of negative expectation effects. Targeted communication strategies, a sensitive use of information in the clarification and positive environmental context conditions can contribute to establishing an adequate expectation management and minimize negative expectation effects in the clinical practice. At the same time, translational research approaches are required to gain further insights into the mediators and moderators of negative expectation effects and to transfer these into clinical practice. In this way the treatment of patients with disorders of the gut-brain communication can be improved.

Die Relevanz von Erwartungseffekten für das Schmerzerleben und die Schmerzbewertung wird zunehmend gewürdigt. Während positive Erwartungseffekte in Forschung wie klinischer Praxis vermehrt Beachtung finden, bleiben die Mechanismen und Konsequenzen negativer Erwartungen weniger gut verstanden. Verfügbare Erkenntnisse stammen zudem meist aus der Erforschung somatischer Schmerzen und sind im Kontext viszeraler Schmerzen trotz profunder Effekte auf die Darm-Gehirn-Achse unzureichend. Ein tieferes Verständnis von Noceboeffekten in der Pathophysiologie viszeraler Schmerzen kann dazu beitragen, negativen Behandlungserwartungen entgegenzuwirken.

In der Alltagssprache häufig als „Bauchgefühle“ bezeichnete Wahrnehmungen umfassen ein großes Spektrum, das von eher als intuitiv empfundenen Gefühlen über diffuse Empfindungen bis hin zu bedrohlich erscheinenden viszeralen Schmerzen reicht. Die neurobiologischen Grundlagen und klinischen Implikationen dieses als Viszerozeption bezeichneten Teilbereichs der Interozeption finden dank neuer Erkenntnisse über die vielfältigen Verbindungen zwischen Darm und Gehirn zunehmend wissenschaftliche Aufmerksamkeit. Interdisziplinäre, translationale Forschungsansätze zur Darm-Gehirn-Achse an der Schnittstelle zwischen Neurogastroenterologie, Schmerzforschung und Neurowissenschaften liefern in diesem Kontext hochrelevante Erkenntnisse bezüglich normaler sowie gestörter Darm-Gehirn-Interaktionen und der Pathophysiologie und Therapie viszeraler Schmerzen. Unter Würdigung eines biopsychosozialen Erkrankungskonzepts ebnet dies den Weg für einen ganzheitlichen Blick auf die Patienten und schafft damit verbesserte Grundlagen für eine personalisierte Therapie.

Negative Erwartungen als Basis für Noceboeffekte sind wissenschaftlich und klinisch unzureichend verstanden

Das Wissen um Erwartungseffekte innerhalb des psychosozialen Behandlungskontexts spezifisch für den viszeralen Schmerz eröffnet ein zukunftsweisendes und spannendes Forschungsfeld, insbesondere für Störungen der Darm-Gehirn-Interaktion wie das Reizdarmsyndrom. Die Bedeutung positiver Therapieerwartungen, gemeinhin als Placeboeffekte bezeichnet, ist dabei bereits vergleichsweise gut untersucht, sowohl in randomisierten, kontrollierten klinischen Studien verschiedener mit Schmerz und viszeralen Beschwerden einhergehender gastrointestinaler Erkrankungen als auch im Hinblick auf das therapeutische Potenzial eines gezielten Einsatzes von Placebointerventionen in der Behandlung von Patienten mit chronischem viszeralem Schmerz [7]. Negative Erwartungen als Grundlage für Noceboeffekte sind hingegen weder wissenschaftlich noch klinisch hinreichend verstanden, und ein Großteil des Wissens bezüglich der Effekte negativer Erwartungen basiert bislang auf Forschungsarbeiten aus der somatischen Schmerzforschung. Ziel dieser Übersichtsarbeit ist es, für den Bereich der viszeralen Schmerzen im Kontext der Darm-Gehirn-Achse aktuelles Wissen und klinische Implikationen bezüglich negativer Erwartungseffekte zusammenzufassen, Forschungslücken aufzuzeigen und Perspektiven für die Translation in die Klinik zu eröffnen.

## Viszeraler Schmerz und die Darm-Gehirn-Achse

Interozeptive, viszerale Schmerzen gehen von den inneren Organen aus und unterscheiden sich qualitativ in vielerlei Hinsicht von exterozeptiven, somatischen Schmerzen. Sie sind nur schwer eindeutig zu lokalisieren, werden häufig von neurovegetativen Symptomen wie Übelkeit, Erbrechen, Schwitzen oder erhöhter Herzrate begleitet und können auf angrenzende Körperareale übertragen werden [1]. Zudem werden sie bereits bei vergleichsweise niedriger Intensität in ihrer emotionalen Qualität auch von Gesunden als unangenehmer empfunden und lösen in stärkerem Maße emotionale Reaktionen wie Furcht aus [14], was die hohe Relevanz psychologischer Faktoren im Kontext des viszeralen Schmerzgeschehens unterstreicht.

Die Kommunikation zwischen Darm und Gehirn verläuft bidirektional

Interessanterweise dokumentieren Hirnbildgebungsstudien, dass die zentralnervösen Prozesse der akuten viszeralen Schmerzwahrnehmung trotz erwartbarer Überlappungen zu anderen Schmerzreizen, insbesondere zu somatischen Schmerzen, aber auch zu nichtnozizeptiven aversiven Stimuli, einzigartige neurale Aktivierungsmuster aufweisen [14, 15, 24]. Vor allem Regionen des Salienznetzwerks, eines neuronalen Netzwerks, das am Erkennen bedeutsamer Reize und an der Integration sensorischer mit emotionalen und kognitiven Informationen beteiligt ist, sind an der Verarbeitung viszeraler Reize stärker beteiligt [11]. Eine mögliche Spezifität der Schmerzverarbeitung und -modulation in Abhängigkeit von der Schmerzlokalisation ist zwar bei Personengruppen mit chronischen Schmerzen noch nicht vergleichend untersucht, es ist jedoch davon auszugehen, dass zumindest ein Teil der Befunde innerhalb spezifischer Gruppen (z. B. Rückenschmerzen) nicht in Gänze auf die viszerale Modalität (z. B. Reizdarmbeschwerden) übertragbar ist.

Grundlage für die viszerale Perzeption und den viszeralen Schmerz ist die Darm-Gehirn-Achse. Die Kommunikation zwischen Darm und Gehirn verläuft bidirektional, wobei neben neuronalen Signalwegen das autonome Nervensystem (primär über den Vagusnerv) sowie neuroendokrine und immunologische Systeme und das Mikrobiom des Darms eine Rolle spielen [17]. Viszerale Wahrnehmungsprozesse werden sowohl direkt im Gehirn durch kognitive und affektive Mechanismen als auch durch absteigende modulatorische Signalwege beeinflusst, wodurch wiederum die Aktivität des Verdauungstrakts verändert werden kann [17]. Bei einer Dysregulation des Darm-Gehirn-Systems kann es daher zu Hypersensitivität und Hyperalgesie in der Wahrnehmung von physiologischen Signalen kommen, was wiederum eine erhöhte Aufmerksamkeit auf viszerale Reize (Hypervigilanz) und Veränderungen der Magen-Darm-Motilität zur Folge haben kann [5, 17]. Dies kann zu Störungen führen, die durch chronische viszerale Schmerzen und veränderte Verdauungsfunktionen charakterisiert sind.

## Chronischer viszeraler Schmerz bei Störungen der Darm-Gehirn-Achse

Die Ursachen für chronische viszerale Schmerzen können vielfältig sein. Andauernde bzw. wiederkehrende viszerale Symptome treten häufig auch ohne eindeutig identifizierbare organische Ursache auf. In diesen Fällen spricht man von funktionellen Magen-Darm-Erkrankungen oder in jüngster Zeit von Störungen der Darm-Gehirn-Interaktion, unter denen das Reizdarmsyndrom die häufigste ist [5]. Für Betroffene bedeuten chronische viszerale Beschwerden, insbesondere der viszerale Schmerz, nicht nur erhebliche Beeinträchtigungen des Alltags und eine Reduktion der Lebensqualität. Es ergeben sich auch Herausforderungen für das Gesundheitssystem als Ganzes sowie für die individuelle Patientenführung und Arzt-Patienten-Kommunikation, bedingt durch eine vermehrte Inanspruchnahme medizinischer Leistungen wie beispielsweise wiederholte Diagnostik und mangelnde Adhärenz, aber auch durch eine hohe Komorbidität mit Affektstörungen [5]. Dies erfordert ein differenziertes diagnostisches und therapeutisches Vorgehen unter Berücksichtigung eines biopsychosozialen Krankheitsmodells und des Wissens über die komplexen Mechanismen der Darm-Gehirn-Achse, insbesondere bezüglich der Rolle psychologischer Einflussfaktoren.

Stress ist ein zentraler psychologischer Einflussfaktor, der im Kontext viszeraler Schmerzen und insbesondere im Zusammenhang mit Reizdarmbeschwerden bereits seit vielen Jahren starke Beachtung findet [16]. Diverse Kommunikationswege der Darm-Gehirn-Achse sind an den engen Zusammenhängen zwischen Stress und viszeraler Symptomatik beteiligt, einschließlich des autonomen Nervensystems, des neuroendokrinen und des Immunsystems sowie des Mikrobioms [16]. So kann Stress nachweislich einerseits zu mikrobiellen Veränderungen führen, andererseits lassen sich durch eine Beeinflussung des Mikrobioms, beispielsweise durch Behandlung mit einem Probiotikum, Stressreaktionen sowie abdominelle Störungen modulieren, was insgesamt die Bidirektionalität der Darm-Gehirn-Achse unterstreicht [16]. Diese Erkenntnisse haben einen Paradigmenwechsel in Bezug darauf eingeleitet, wie funktionelle Magen-Darm-Erkrankungen konzeptualisiert werden, nämlich von einer rein peripheren Störung der Darmmotilität ohne organische Marker zu einer Erkrankung, die durch komplexe Dysfunktionen der Darm-Gehirn-Interaktion mit peripheren und zentralnervösen Komponenten gekennzeichnet ist [17].

Dysfunktionale Kognitionen können zur Aufrechterhaltung und Verschlimmerung von Symptomen beitragen

Neben Stress spielen auch maladaptive Kognitionen eine wichtige Rolle [12]. So können bei Patienten mit Reizdarmsyndrom dysfunktionale Kognitionen wie selektive Aufmerksamkeit und Katastrophisieren in Reaktion auf viszerale Signale zur Aufrechterhaltung und Verschlimmerung von Symptomen beitragen und sind mit der Symptomschwere assoziiert. Auch Furcht, negative Vorerfahrungen und affektive Störungen mit Symptomen der Angst und Depression stehen in direktem Zusammenhang nicht nur mit maladaptiven Kognitionen, sondern auch mit einer veränderten viszeralen Schmerzwahrnehmung [12]. Es ist daher von zentraler Bedeutung, auch diese Faktoren der Schmerzmodulation in den Blick zu nehmen, die, wie im Folgenden erläutert, vermutlich essenziell auch an negativen Erwartungseffekten beteiligt sind.

## Erwartungseffekte im psychosozialen Behandlungskontext

Im psychosozialen Behandlungskontext können Erwartungen die Symptom- bzw. Schmerzwahrnehmung sowie das Erleben und Erinnern belastender Symptome stark beeinflussen. Für den Bereich der gastrointestinalen Erkrankungen liegen inzwischen zahlreiche randomisierte, kontrollierte Studien vor, die analog zu vielen anderen Erkrankungen die Bedeutung positiver Therapieerwartungen unterstreichen [7]. Eine aktuelle klinische Studie bei Patienten mit Reizdarmsyndrom erweitert die Datenlage und dokumentiert eine substanzielle Symptomverbesserung nach der Gabe von Placebotabletten, unabhängig davon, ob die Teilnehmer von einem pharmakologischen Wirkstoff ausgingen oder explizit darüber aufgeklärt wurden, dass es sich um wirkstofffreie Placebos handelte (Open-label-Placebo-Behandlung; [19]).

Im Gegensatz zu solchen Effekten positiver Therapieerwartung rückt die Bedeutung negativer Erwartungen und somit potenziell schmerzamplifizierender Noceboeffekte erst langsam in den Fokus systematischer Untersuchungen, obgleich diese im Behandlungskontext höchst relevant sind und den Behandlungsverlauf und -erfolg maßgeblich beeinflussen können. Daten aus den an die Viszeralmedizin angrenzenden Disziplinen zeigen, dass schätzungsweise 40–100 % der Nebenwirkungen von Medikamenten nicht auf den Wirkstoff selbst, sondern auf den Behandlungskontext zurückzuführen sind [20]. Negative Erwartungseffekte können zudem die Wirksamkeit therapeutischer Interventionen beeinträchtigen [3]. Für den viszeralen Schmerz konnte bereits 1987 dokumentiert werden, dass die Beschreibung möglicher gastrointestinaler Nebenwirkungen während des Einwilligungsgesprächs zu einem 6fachen Anstieg der berichteten gastrointestinalen Nebenwirkungen führte [23]. Da „Nocebo“ ursprünglich jedoch vor allem unerwünschte Ereignisse beschrieb, die von Studienteilnehmern nach Gabe eines Placebopräparats berichtet wurden [7], und sehr frühe experimentelle Nachweise die Begrifflichkeit „Nocebo“ noch nicht verwendeten (für einen kurzen historischen Abriss siehe [7]), wurde der Einfluss negativer Erwartung, insbesondere aber auch seine klinischen Implikationen, möglicherweise in der Wissenschaft und öffentlichen Wahrnehmung lange unterschätzt. In einer neueren prospektiven Studie sagten jedoch einzig psychosoziale Faktoren, vor allem die Erwartung eines langwierigen und komplikationsbehafteten Heilungsprozesses, die Entwicklung eines Reizdarmsyndroms nach einem gynäkologischen Eingriff vorher [30]. Seither wächst auch das Interesse an psychologischen Mechanismen, die der Entstehung und Aufrechterhaltung negativer Erwartungseffekte bei viszeralen Schmerzen zugrunde liegen.

## Psychologische Mechanismen negativer Erwartungseffekte

**Figure 1:**
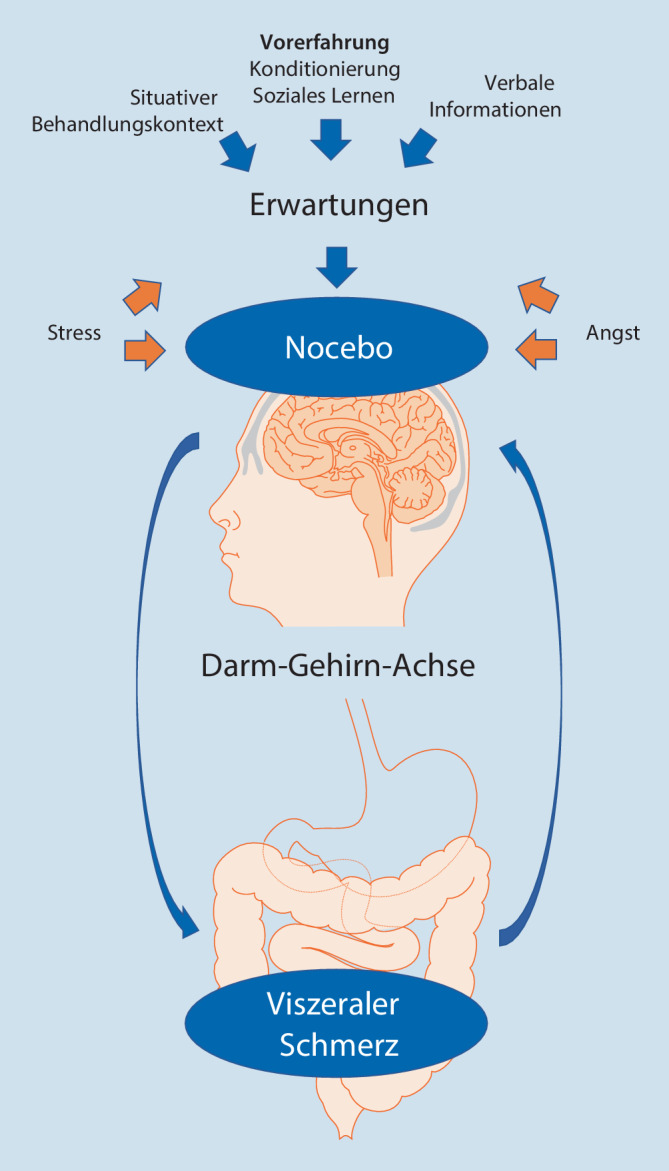

Aktuelle konzeptionelle Ansätze gehen von verschiedenen, aber nicht zwingend unabhängigen psychologischen Mechanismen aus, die Noceboeffekten zugrunde liegen (Abb. 1). Dazu gehören insbesondere Faktoren des Behandlungskontexts, vor allem die durch Behandler vermittelten Informationen und Instruktionen sowie individuelle Therapieerfahrungen und Lernprozesse. Laut einer Metaanalyse scheint besonders die Kombination von verbaler Instruktion und Lernprozessen starke hyperalgetische Effekte induzieren zu können [26], wobei dies bislang nicht speziell für viszerale Schmerzen nachgewiesen werden konnte [8].

An einem experimentellen viszeralen Schmerzmodell wurde beobachtet, dass das Erleben und die neuronale Verarbeitung viszeraler Schmerzen allein durch verbale Informationen negativ beeinflussbar sind [28, 29]. Dabei wurde bei gesunden Probanden eine negative Behandlungserwartung durch die Information induziert, einen den Akutschmerz kurzfristig verstärkenden Opioidantagonisten zu erhalten, obgleich die tatsächliche Behandlung lediglich mit einer wirkstofffreien Kochsalzlösung (hier: einem Nocebo) erfolgte. Veränderungen der viszeralen Schmerzbewertung sowie der neuronalen Verarbeitung viszeraler Schmerzreize wurden mithilfe eines rektalen Dehnungsmodells untersucht, mit dem häufige Symptome bei Störungen der Darm-Gehirn-Achse auch bei Gesunden valide simuliert werden können. Negative Behandlungserwartungen verstärkten in diesem experimentellen Ansatz sowohl die Schmerzerwartung und -wahrnehmung als auch die aversive Empfindung des Stuhldrangs – ein Effekt der durch experimentell induzierten Stress gesteigert wurde [28] – und waren mit Veränderungen der Schmerzverarbeitung in der Insula, dem Thalamus und der Amygdala assoziiert [29]. Jedoch sind diese Daten bislang ausschließlich bei gesunden Probanden erhoben worden und können nicht ohne Weiteres auf Schmerzpopulationen übertragen werden.

Frühere Schmerzerfahrungen und die Schmerzbehandlungshistorie tragen nachweislich zu negativen Erwartungseffekten bei [25], was die Rolle des Lernens im Kontext von Noceboeffekten unterstreicht. Insbesondere Furcht vor viszeralen Schmerzen kann durch Lernen entstehen und ist dynamischen Veränderungen unterworfen. Sie trägt zu erhöhtem Stress, negativen Affekten und maladaptiven Kognitionen bei und ist – in Übereinstimmung mit zentralen Annahmen von Furcht-Vermeidungs-Modellen des chronischen Schmerzes – fundamental an der Aufrechterhaltung von Schmerzen beteiligt [22]. Experimentelle Arbeiten zu viszeralen schmerzbezogenen Lernprozessen bei Gesunden und Patienten mit Reizdarmsyndrom dokumentieren systematisch den Einfluss klassischer Konditionierung auf Erwartungseffekte im Kontext der Darm-Gehirn-Achse [6]. Durch klassische Konditionierung induzierte Veränderungen der Schmerzantizipation können dabei die zentralnervöse Verarbeitung viszeraler schmerzhafter, aber auch nichtschmerzhafter Symptome verändern, insbesondere wenn diese als unvorhersehbar erlebt werden [6], das heißt, wenn es keine spezifischen Hinweise gibt, die das Auftreten von Schmerzen vorhersagen, oder kein Zusammenhang zwischen spezifischen Hinweisen und Schmerzen erkannt wird [22], was experimentell anhand von Bewertungen der Kontingenz zwischen prädiktiven Reizen und Schmerzstimuli quantifiziert werden kann.

Frühere Schmerzerfahrungen und -behandlungen tragen zu negativen Erwartungseffekten bei

Im Bereich somatischer Schmerzen ist gut belegt, dass Unvorhersehbarkeit Hyperalgesie fördern kann [22], sodass dies auch bei Patienten mit viszeraler Symptomatik, die oft mit unvorhersehbaren Schmerzen konfrontiert sind, zu einer Verschlimmerung ihrer Symptome beitragen könnte. Interessant sind in diesem Zusammenhang auch erste Belege dafür, dass akuter Stress negative Erwartungseffekte bei Gesunden offenbar verstärkt [28]. Da sowohl die viszerale Schmerzsensitivität als auch das differenzielle schmerzbezogene Lernen durch das Stresshormon Kortisol modifizierbar sind [2] und der Erwerb schmerzbezogener Furcht bei Patienten mit Reizdarmsyndrom verändert ist [10], erscheinen psychologische Faktoren wie Stress und (erlernte) Furcht als Amplifikatoren des Noceboeffekts insbesondere in Bezug auf viszerale Schmerzen naheliegend. Aktuelle Daten zeigen zudem, dass eine Aktivierung des zentralnervösen Furchtnetzwerks auch durch harmlose interozeptive „Bauchgefühle“ evoziert werden kann, wenn diese als erlernte Prädiktoren viszeraler Schmerzen wahrgenommen werden [11]. Bei derartigen interozeptiven Lern- und Gedächtnisprozessen scheinen kontextuelle Faktoren und Veränderungen der Aufmerksamkeit maßgeblich beteiligt zu sein. Diese Befunde unterstreichen die Relevanz interozeptiver Konditionierung in der Pathophysiologie viszeraler Schmerzen mit klaren Implikationen für negative Erwartungseffekte im Behandlungskontext, bei denen interozeptiver Hypervigilanz eine zentrale Rolle zukommen könnte. Insgesamt bilden Modelle schmerzbezogener Lern- und Gedächtnisprozesse wertvolle translationale Ansätze und liefern in Form des Extinktionslernens auch die konzeptionelle Basis für expositionstherapeutische Ansätze bei chronischem viszeralem Schmerz, die Furcht und negative Erwartungen reduzieren und positive Effekte auf die Schmerzsymptomatik haben können [6].

## Implikationen für die Therapie chronischer viszeraler Schmerzen

Aus Erkenntnissen über negative Erwartungseffekte lassen sich verschiedene Implikationen für die Optimierung der Behandlung von Patienten mit chronischen viszeralen Schmerzen ableiten. Dabei sollten der Behandlungskontext, die Kommunikation und modulierende psychologische Faktoren gleichermaßen in den Fokus genommen werden. Patienten mit chronischen viszeralen Schmerzen haben oft negative Vorerfahrungen im Behandlungskontext gemacht, etwa in der Kommunikation oder mit früheren Behandlungsversuchen. Es liegt nahe, dass gerade bei sogenannten Ausschlussdiagnosen wie dem Reizdarmsyndrom, für das nach teils aufwendiger Diagnostik zudem keine eindeutige, erfolgreiche Therapiestrategie existiert, das Risiko für negative Vorerfahrungen besonders hoch ist. Dadurch geprägte Erwartungen und Ängste im Vorfeld zu identifizieren, kann dazu beitragen, die Arzt-Patienten-Beziehung zu optimieren und durch frühere negative Erfahrungen induzierten Noceboeffekten entgegenzuwirken [13]. Neben einer auf den Patienten abgestimmten Aufklärung über den Einfluss von Noceboeffekten im Behandlungskontext [9] ermöglichen multimodale Ansätze in der Schmerztherapie es, Stress, erlernte Furcht, maladaptive Kognitionen und Vermeidungsstrategien als Amplifikatoren negativer Erwartungseffekte in den Fokus zu rücken [22] und adäquate Bewältigungsstrategien zu vermitteln, um Noceboeffekte effizient zu reduzieren [21].

Eine verbesserte Kommunikation kann die Patientenerwartung positiv beeinflussen

Erkrankungsübergreifende Konzepte machen deutlich, dass die Patientenerwartung durch eine verbesserte Kommunikation seitens aller am Behandlungsprozess Beteiligten positiv beeinflusst werden kann [21]. So kann zur Vermeidung negativer Erwartungseffekte die informierte Einwilligung angepasst werden, unter anderem lassen sich Angaben zu Nebenwirkungen positiv formulieren (Beispiel: „95 % der Betroffenen haben keine Probleme …“; [27]). Ein Verzicht auf Informationen über Nebenwirkungen erzielt zwar positive Ergebnisse, muss allerdings ethisch und berufsrechtlich diskutiert werden [31]. Neue Wege zur Vermeidung negativer Erwartungseffekte unter Beibehaltung der informierten Zustimmung werden derzeit erarbeitet [31]. Eine gezielte Schulung und Sensibilisierung medizinischen Personals sowohl zur Relevanz von Erwartungseffekten als auch zur Vermeidung von Noceboeffekten wird als zentraler Faktor betrachtet [9]. Dabei sollten nicht nur die verbale Kommunikation, sondern auch nonverbale Verhaltensweisen wie Blickkontakt und empathische Zuwendung Berücksichtigung finden, die sich nachweislich auf das Schmerzerleben auswirken können [4]. Zentral und bereits systematisch integriert [18] ist die Anerkennung, dass eine Optimierung der Arzt-Patienten-Kommunikation insbesondere bei Patienten mit Störungen der Darm-Gehirn-Interaktion eine tragende Säule jeglicher Diagnostik und Therapie sein sollte.

## Fazit für die Praxis

- Negative Erwartungseffekte beim viszeralen Schmerz können durch verbale Instruktionen, den Behandlungskontext und Lernprozesse induziert und moduliert werden.
- Psychologische Faktoren wie maladaptive Kognitionen, Angst und Stress sind essenziell an negativen Erwartungseffekten beteiligt und können sich auch direkt auf die viszerale Symptomatik auswirken.
- Bei der Gestaltung einer tragfähigen und vertrauensvollen Arzt-Patienten-Beziehung und zur Reduktion negativer Erwartungseffekte sollten im Rahmen multimodaler Ansätze der Behandlungskontext, die Kommunikation und modulierende psychologische Faktoren gleichermaßen in den Fokus genommen werden.
- Eine Optimierung der Arzt-Patienten-Kommunikation, insbesondere bei Patienten mit Störungen der Darm-Gehirn-Interaktion, sollte eine tragende Säule jeglicher Diagnostik und Therapie sein.
